# Genetic Polymorphisms Complicate COVID-19 Therapy: Pivotal Role of HO-1 in Cytokine Storm

**DOI:** 10.3390/antiox9070636

**Published:** 2020-07-18

**Authors:** Eddie W. Fakhouri, Stephen J. Peterson, Janish Kothari, Ragin Alex, Joseph I. Shapiro, Nader G. Abraham

**Affiliations:** 1 New York Presbyterian Brooklyn Methodist Hospital, Brooklyn, NY 11215, USA; edf9027@nyp.org (E.W.F.); jmk9029@nyp.org (J.K.); 2 Department of Medicine, Weill Cornell Medicine, New York, NY 10065, USA; 3 Department of Pharmacology, New York Medical College, Valhalla, NY 10595, USA; ralex@nymc.edu; 4 Joan C. Edwards School of Medicine, Marshall University, Huntington, WV 25701, USA; shapiroj@marshall.edu; 5 Department of Medicine, New York Medical College, Valhalla, New York, NY 10595, USA

**Keywords:** SARS-CoV-2, COVID-19, cytokine storm, HO-1, ACE-2, ACE-2R, Genetic polymorphisms, Cytochrome P450, CYP2D6, mitochondrial dysfunction, white adipose tissue

## Abstract

Coronaviruses are very large RNA viruses that originate in animal reservoirs and include severe acute respiratory distress syndrome (SARS) and Middle East respiratory syndrome (MERS) and other inconsequential coronaviruses from human reservoirs like the common cold. SARS-CoV-2, the virus that causes COVID-19 and is believed to originate from bat, quickly spread into a global pandemic. This RNA virus has a special affinity for porphyrins. It invades the cell at the angiotensin converting enzyme-2 (ACE-2) receptor and binds to hemoproteins, resulting in a severe systemic inflammatory response, particularly in high ACE-2 organs like the lungs, heart, and kidney, resulting in systemic disease. The inflammatory response manifested by increased cytokine levels and reactive oxygen species results in inhibition of heme oxygenase (HO-1), with a subsequent loss of cytoprotection. This has been seen in other viral illness like human immunodeficiency virus (HIV), Ebola, and SARS/MERS. There are a number of medications that have been tried with some showing early clinical promise. This illness disproportionately affects patients with obesity, a chronic inflammatory disease with a baseline excess of cytokines. The majority of the medications used in the treatment of COVID-19 are metabolized by cytochrome P450 (CYP) enzymes, primarily CYP2D6. This is further complicated by genetic polymorphisms of CYP2D6, HO-1, ACE, and ACE-2. There is a potential role for HO-1 upregulation to treat/prevent cytokine storm. Current therapy must focus on antivirals and heme oxygenase upregulation. Vaccine development will be the only magic bullet.

## 1. Introduction

SARS-CoV-2 is a betacoronavirus, an RNA virus that shares the same subgenus as severe acute respiratory distress syndrome (SARS) [1]. Human coronaviruses include the common cold and are generally inconsequential. Both SARS and Middle East respiratory syndrome (MERS) came from animal reservoirs and caused global epidemics. Coronaviruses are large viruses and include alpha, beta, delta, and gamma subgroups, with alpha and beta affecting humans [2]. The early presenting signs and symptoms of the coronavirus are very non-specific [3]. SARS-CoV-2, the virus that causes COVID-19, has a tremendous ability to bind porphyrins with even stronger affinity than ACE-2 receptors, resulting in the upregulation of free heme, an oxidant, and severe reactive oxygen species (ROS) formation, and decreased levels of heme oxygenase-1 enzymes (HO-1) [4]. Some have found these assertions controversial [5] but these findings may provide insight into the pathophysiologic process occurring with COVID-19 infection and possible treatment options.

The HO system, which comprises HO-1 (inducible) and HO-2 (constitutive), which degrade heme to free iron (increased ferritin synthesis), bilirubin (antioxidant), and carbon monoxide (antiapoptotic) is critical in its primary role in cellular defense is involved in a variety of crucial physiological functions. These include cytoprotection, anti-inflammation, anti-oxidative effects, apoptosis, neuromodulation, immune-modulation, angiogenesis, and vascular regulation [6]. Increased levels of HO-1 expression also results from numerous forms of stress, including smoking, cytokines, hypoxia, heme, oxidative stress, heat shock, ROS, nitric oxide (NO), cAMP, and oxidized lipids [6].

## 2. COVID-19 Increases Free Heme and Decreases Functional Hemoprotein

**Figure 1:**
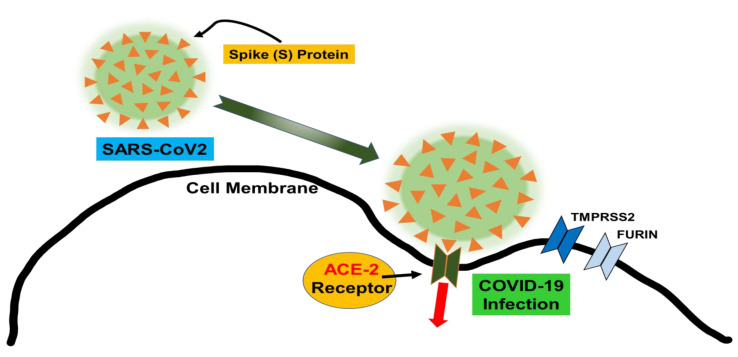
This represents the spike protein of the COVD-19 virus. The spike enters the cell through the ACE-2 receptor. The spike is subsequently cleaved by the following proteases, transmembrane serine protease 2 (TMPRSS2), and FURIN, creating an active COVID-19 infection.

COVID-19 produces a severe systemic inflammatory reaction, likely the result of increased free heme levels and decreased levels of HO-1 activity and functional hemoprotein. The spike protein of COVID-19 binds ACE-2 receptors (Figure 1) and porphyrin molecules with weak and strong affinity, respectively [7]. Porphyrins are the building blocks of heme and allow COVID-19 access to invade cells along with ACE-2 receptors and bind functional hemoprotein within the cell [7]. The resulting COVID-porphyrin complex may lead to an impairment of HO-1 function and an upregulation of pro-inflammatory free heme and iron, which overwhelms the anti-inflammatory cytoprotection of HO-1 [6].

**Figure 2:**
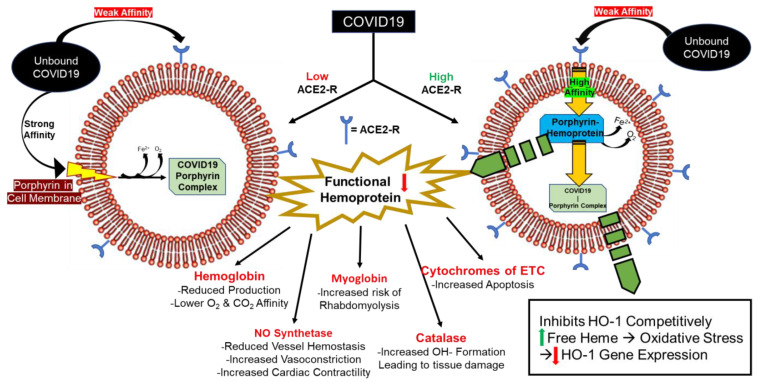
COVID-19 infects the cells. COVID-19 increases free heme and free iron (Fe), but more importantly, decreases functional hemoprotein. (**Left**) Unbound COVID-19 binds to porphyrin within the cell membrane with high affinity, providing entry into the cell. Iron and oxygen are released forming a COVID-19-Porphyrin complex. The COVID-19-porphyrin complex competitively inhibits heme oxygenase (HO-1) leading to severe oxidative stress from free heme and iron, and subsequently downregulates HO-1 gene expression. (**Right**) Unbound COVID-19 binds ACE-2 receptor with lower affinity than porphyrin, and upon internalization into the cell, binds to the porphyrin substrate of hemoprotein within the cell. The result is a malfunctional hemoprotein, and with the release of iron and oxygen, a COVID-19-porphyrin complex. The consequences of increased free heme and iron along with decreased HO-1 levels, inhibition of HO-1, and decreased functional hemoprotein levels have detrimental effects to the infected cells. Key: oxygen = O_2_, carbon dioxide = CO_2_, electron transport chain = ETC, hydroxide = OH^−^.

COVID-19 infects cells with low ACE-2 receptor concentration [low ACE-2R] and high [ACE-2R] with worse outcomes occurring in high [ACE-2R] cells because COVID-19 has a high affinity for the porphyrin substrate on hemoprotein within the cell, which causes a decrease in functional intracellular hemoprotein levels (Figure 2). Thus, COVID-19 produces clinical signs and symptoms mainly affecting high [ACE-2R] organs such as the lungs (endothelium), kidney, liver, gastrointestinal (GI) tract, testes, and heart [8].

## 3. How COVID-19-Cytokine Storm Inhibits HO-1 

**Figure 3:**
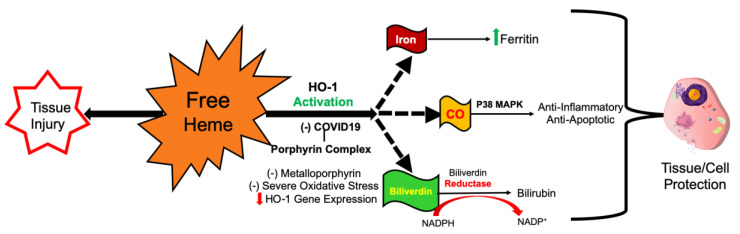
As more COVID-19-Porphyrin complexes are being produced the competitive inhibition increases, eventually out-competing heme leading to increasing reactive oxygen species (ROS) formation. The result is less production of ferritin, Fe, bilirubin, and carbon monoxide (CO). This may be part of the reason why COVID-19 patients have fluctuations in ferritin and bilirubin, which are measurable inflammatory markers. Furthermore, free iron release from the binding of COVID-19 to porphyrin increases ferritin even when HO-1 is inhibited. The end result is increased free heme and iron that leads to severe oxidative stress causing decreased HO-1 gene expression and competitively inhibited HO-1 enzymes that were previously active. Key: p38 mitogen-activated protein kinases = P38 MAPK, nicotinamide adenine dinucleotide phosphate (NADP^+^/NADPH).

Inhibitors of HO activity are metalloporphyrins, which do not carry an iron molecule but are replaced by tin, chromium, or zinc, inhibit the rate limiting enzyme heme oxygenase [9,10,11,12,13,14,15]. Tin (Sn^4+^) has been used to lower bilirubin levels in newborns to treat hyperbilirubinemia in newborns [6]. HO-1 cleaves the tetrapyrrole ring at the methylene bridge to produce carbon monoxide (CO) biliverdin, and iron [6,16]. Biliverdin is reduced to bilirubin by biliverdin reductase in mammals. Since COVID-19 binds porphyrin and removes iron and oxygen to form a COVID-19-Porphyrin complex, it can behave as a metalloporphyrin and competitively inhibits HO-1 (Figure 3) [17].

## 4. COVID-19 Induction of ALAS-1/2

**Figure 4:**
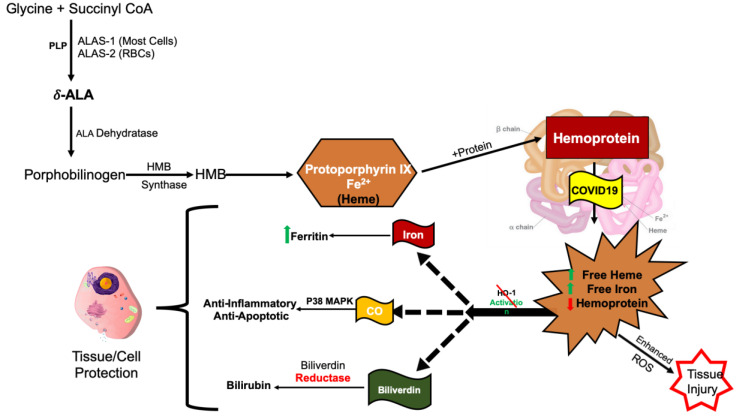
The porphyrin synthesis pathway in relation to COVID-19. The porphyrin synthesis pathway displaying COVID-19 binding the end product, hemoprotein, which binds non-enzymatically to porphyrin causing upregulation of heme production as a result of negative feedback due to a decrease in functional hemoprotein. This leads to increased free heme, decreased functional hemoprotein, and the release of toxic iron, resulting in tissue injury. Key: CoA = coenzyme A, ALA = delta-aminolevulinic acid, and HMB = hydroxymethylbilane.

The COVID-porphyrin complex leads to an upregulation of pro-inflammatory free heme production presumably by inducing delta-aminolevulinic acid (ALAS-1/2), the first step and rate limiting enzyme in the synthesis of the heme pathway [18]. Since COVID-19 is able to bind heme at the porphyrin binding site, it reduces the amount of functional hemoprotein, potentially inducing ALAS-1/2 to produce more heme as a result of a negative feedback loop (Figure 4). ALA synthetase and porphobilinogen deaminase (PBGD), which are present in both adipocytes and red cells maintain heme levels. ALAS-1/2 increases heme degradation by HO-1 [19]. COVID-19 produces dysfunctional hemoproteins and dysfunctional porphyrin that are no longer capable of making heme. This leads to more hemoprotein available for COVID-19 to bind to, which leads to the release of more free iron, and as a result, increased inflammation [6]. In addition, the iron released by dying cells has additive toxic effects. Murine models of HO-1 deficiency demonstrate a loss of stress defenses and increased toxicity from free iron [20,21,22]. Humans with HO-1 deficiency resulted in death at an early age [23,24], as a result of lack of stress defenses [25], tubular injury [24], hemolysis, nephritis, and asplenia [26]. The increased inflammation leads to severe oxidative stress which further decreases HO-1 gene expression [6]. Drugs currently being used to treat COVID-19 patients include Chloroquine, Hydroxychloroquine, Azithromycin, and Remdesivir are all cytochrome P450 (CYP) inhibitors [27], although Remdesivir has only shown this for in vitro activity by Gilead Sciences as described in the Remdesivir (GS-5734) investigator’s brochure, Edition 5, 21 February 2020 and listed in the NIH COVID-19 Treatment Guidelines, Since these drugs downregulate CYP, which has a heme moiety, this decreases the demand for heme production [28]. The success of these drugs is likely attributable to the fact that they all inhibit CYP production which results in lower heme production [29].

## 5. Consequences of Hemoprotein Malfunction/Deficiency

The consequence of COVID-19 binding the porphyrin molecules results in decreased functional hemoprotein, which has clinical manifestations [4]. A hemoprotein is a protein linked to iron (Fe). The Fe group attached to the protein is capable of undergoing oxidation reduction (redox) reactions and function within the electron transport chain (ETC) of the mitochondria. Hemoproteins include hemoglobin, myoglobin, nitric oxide (NO) synthetase, catalase, and the cytochromes of the ETC in the mitochondria. Deficiency of any of these hemoproteins cause tissue inflammation and organ damage and even a prothrombotic state (Table 1). Many COVID-19 patients require prolonged periods of supplemental oxygenation to maintain saturation levels even after all other symptoms have resolved. This is due to the decreased oxygen affinity COVID-19 causes in hemoglobin leading to the displacement of iron [7]. Deficiency of human HO-1 causes early death as a result of severe iron toxicity, renal failure, and nephrotic range proteinuria, the same clinical issues we have faced with COVID-19 [23,24,25,26,30]. Similar to hemoglobin, myoglobin is also affected by COVID-19. A significant number of COVID-19 patients experience rhabdomyolysis due to COVID-19 directly binding the porphyrin substrate of myoglobin, leading to the release of hemoprotein and iron, which induces inflammation and muscle breakdown [31,32,33,34]. This has previously been shown in HO-1 deficiency in vitro and in vivo [35,36,37]. It has been proposed that the severe inflammation in COVID-19 downregulates HO-1 further exacerbating the severe inflammatory response [17]. 

NO synthetase is a hemoprotein probably affected by COVID-19. If COVID-19 impairs NO synthetase, NO levels decrease. Recent studies have tried using inhaled nitric oxide to mitigate the severity of COVID-19 to supplement the amount normally produced by the nasal turbinates to promote bronchodilatory and vasodilatory effects [38,39,40]. Previous studies have demonstrated patients who have an impaired NO pathway include those with hypertension, diabetes mellitus, and atherosclerosis, patients observed to have worse outcomes from COVID-19 infection [41]. In the kidney, NO functions to regulate renal hemodynamics, medullary perfusion, natriuresis, tubuloglomerular feedback, inhibition of tubular sodium reabsorption, and modulation of renal sympathetic neural activity [42]. Endothelial cells produce NO to compensate for the growing demand for oxygen and to provide anti-inflammatory and healing actions in the vasculature [6]. In contrast, vasoconstrictors, such as endothelin 1, angiotensin II (ANG-II), and thromboxane A, act as opposing molecules to nitric oxide (NO) [6].

Cytochrome P450 (CYP) enzymes are hemoproteins [43,44]. As a result, if CYP enzymes levels increase, heme production is increased [28]. Alternatively, if CYP enzymes are decreased, the result is decreased heme production [28]. Decreased CYP production decreases heme production and decreases inflammation. Drugs used to treat COVID-19 are CYP inhibitors, which include Azithromycin, Lopinavir, Hydroxychloroquine, and Chloroquine [27].

Catalase and cytochrome enzymes require heme. Catalase is a vital hemoprotein also potentially affected by COVID-19 as its malfunction would be responsible for directly increasing free ROS. When catalase is not present, the conversion of hydrogen peroxide (H_2_O_2_) to water (H_2_O) and O_2_ does not occur [44]. Cytochromes in the ETC, including cytochrome C, are also hemoproteins potentially affected by COVID-19. The binding of COVID-19 may cause dysfunction of the ETC in cells and may translocate cytochrome C into the cytosol from mitochondria, which induces an apoptosis cascade [45].

## 6. HO-1 Genetic Polymorphisms and COVID-19′s Cytokine Storm

It has been demonstrated in multiple studies that HO-1 genetic polymorphisms, specifically the GT dinucleotide repeat in the promoter region, regulates the inducibility (i.e., transcription) of HO-1 to ROS [46,47,48,49,50,51,52]. Individuals with larger GT repeats have been found to be more susceptible to diseases that involve the endothelium of the cardiovascular system including abdominal aortic aneurysms, atherosclerosis, and coronary artery disease, especially in diabetes and obesity [46,49,50]. Other diseases such as emphysema and melanoma were also more prevalent in patients with larger GT repeat sequences [47,48]. Whether or not shorter GT alleles are associated with higher levels of HO-1 and better glycemic control remain controversial, but there is agreement that if any correlation is suspected, the shorter GT allele is associated with mild beneficial glycemic control [52,53].

HO-1 levels are lower in those with longer GT sequences predisposing patients to more inflammation and decreased endothelial hemostasis [46,47,48,49,50,51,52]. Individuals with long GT repeats had lower bilirubin and ferritin levels [54], reviewed in [55], and in vitro studies displayed increased HO-1 levels when induced by oxidative stress in lymphoblastoid cell lines with shorter GT sequences than in those with longer GT sequences [49,52]. The result was increased oxidant-induced apoptosis in lymphoblastoid cell lines with longer GT sequences [49]. That is, short alleles of the GT repeat are associated with greater inducibility of HO-1, which leads to increased cytoprotection and reduced inflammation [52].

**Figure 5:**
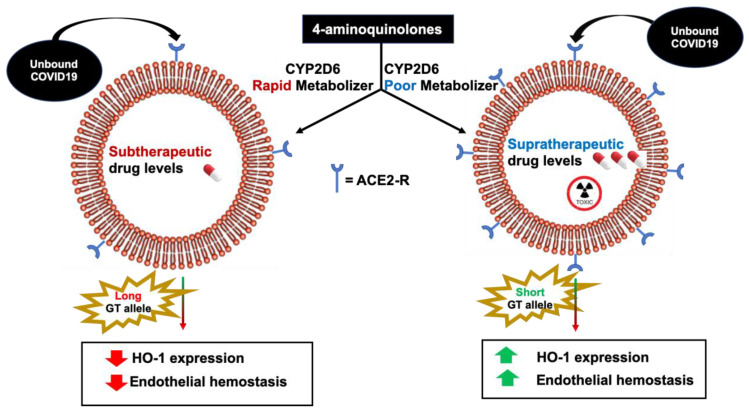
(**Left**) Individuals with the longer GT repeat allele HO-1 genetic polymorphism has lower HO-1 expression and decreased endothelial hemostasis. Rapid CYP2D6 metabolizers develop subtherapeutic drug levels. (**Right**) Individuals with the shorter GT repeat allele have higher HO-1 expression and increased endothelial hemostasis. Poor CYP2D6 metabolizers develop toxic drug levels. The endothelium in all organs can be affected in larger GT allele individuals including the endothelium in the lungs, kidney, and the rest of the cardiovascular system.

Perhaps COVID-19 patients with longer GT sequences are at higher risk of developing the complications of COVID-19 including decreased vessel hemostasis (Figure 5). Further research needs to be done to determine if there is a correlation. Patients with Gilbert syndrome are known to have shorter GT sequences and elevated HO-1 levels and bilirubin [46,56,57,58]. These patients are well protected from cardiovascular disease due to a lower inflammatory state given the increase in bilirubin and HO-1 levels at baseline and during times of stress (e.g., fasting), which decreases ROS [46,56,57,58]. These findings further support that shorter GT sequences lead to a lower inflammatory state.

COVID-19 has worse outcomes in the obese and diabetic populations, likely because these patients are already in a proinflammatory state at baseline due to elevated levels of interleukin 6 (IL-6) from insulin and leptin resistance [59,60]. Obese and metabolic syndrome patients have both insulin and leptin resistance leading to appetite stimulation further exacerbating rises in insulin and leptin levels [59,61]. As a result, IL-6 is expressed and released from adipocytes (mainly white fat cells) through multiple signal pathways resulting in inflammation [62]. Insulin resistance also results in chronic very low density lipoprotein (VLDL) secretion and increased delivery of acyl moieties to muscle, which if provided in excess, will lead to induction of insulin resistance irrespective of obesity [63].

There are many reasons for the negative clinical outcomes in the chronic inflammatory state of obesity as result of COVID-19 [60]. Peterson et al. have shown that obesity increases the oxidation of high-density lipoprotein (HDL) [64]. Oxidized HDL (Ox-HDL) alone is considered to potentiate increased inflammatory cytokines levels by the direct effect on adipocyte stem cells [65]. Additionally, Ox-HDL causes an inflammatory cascade with inflammatory cytokines, interleukins (IL-6, IL-1), tumor necrosis factor (TNF), and upregulation of Angiotensin II (ANG II), a biomarker for early detection of cardiovascular risk [64]. This made obese subjects susceptible to early heart failure and death subsequent to COVID-19 infection [66]. We showed that humans and mice with obesity display low left ventricle function [67]. In a study of inflammatory markers comparing obese women in rural Appalachia to women in urban Brooklyn, Brooklyn had higher levels of both IL-6 and TNF, but both had low levels of adiponectin, marked elevation of Ox-HDL, and circulating endothelial cells (CEC). Obesity increases the volume of epicardial fat and subsequent inflammation converts beige fat to white fat after uncoupling of mitochondrial enzymes and destruction of mitochondria [68]. This negatively affected cardiovascular outcomes in obesity including heart failure and arrhythmias, even prior to COVID-19 infection. Increased antioxidant and HO-1-derived bilirubin may ameliorate the negative effect of COVID-19. Recent studies showed that upregulation of HO-1 attenuated this risk [68], and converts white epicardial fat to preferred beige epicardial fat [68,69]. Increasing HO-1 levels with pharmacological therapy [70] may be with an advantageous effect in severe inflammation states.

**Figure 6:**
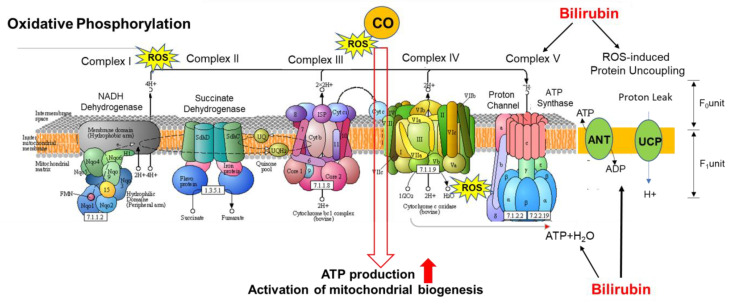
As ATPase activity increases, more electrons and protons permeate through the mitochondrial membrane into the matrix, leading to the formation of ROS. Molecules such as uncoupling proteins (UCPs), and adenine nucleotide translocases (ANTs) uncouple these reactions counteracting ATPase’s increased activity lowering the formation of ROS with increased thermogenesis. Furthermore, the ROS formed by COVID-19′s “cytokine storm” create inflammation that is destructive to the mitochondria in the electron transport chain (ETC). If insufficient mitochondria in cells is evident, such as in white adipose cells, these cells are unable to accommodate the severe ROS formed leading to overwhelming inflammation. Brown adipose cells are better at handling ROS due to higher concentrations of mitochondria. CO increases ATP production and activates mitochondrial biogenesis [85,86,87] and bilirubin blocks ROS action and increases resistance to hepatic steatosis. [88,89].

**Figure 7:**
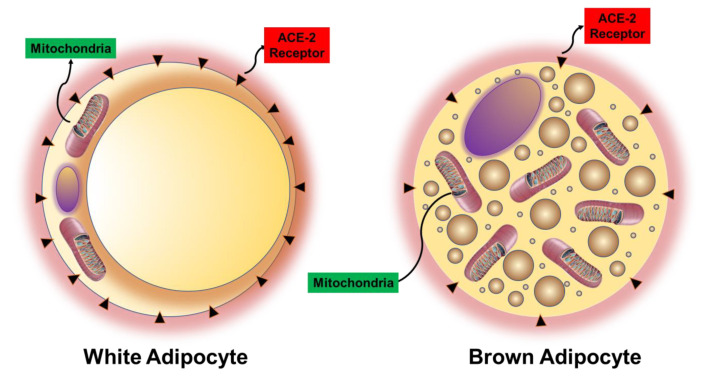
ACE-2 is overexpressed in adipose tissue in obesity and diabetes allowing COVID-19 to infect the cell. Lean (brown adipocyte) tissue is characterized by high amounts of mitochondria and HO-1 levels which result in low levels of ROS. Brown adipose tissue is better than white adipose tissue in its ability to handle the inflammation cause by COVID-19. Obese (white adipocyte) tissue is characterized by low amounts of mitochondria and HO-1, which results in high levels of ROS and upregulation of ACE-2 receptors, especially in morbid obesity and diabetes. COVID-19 patients with high amounts of white adipose tissue develop severe inflammation.

The obese population also has a higher distribution of white fat relative to non-obese people, who have higher amounts of brown fat [71]. White fat is proinflammatory and has lower concentrations of mitochondria than brown fat [72]. Mitochondria is a major source of ROS. ROS generated in the mitochondria occurs at the ETC during the process of oxidative phosphorylation [64,67] (Figure 6). During oxidative phosphorylation energy is transferred through four multi protein complexes (I–IV) embedded in the inner mitochondrial membrane to create adenosine triphosphate (ATP) cofactors, nicotinamide adenine dinucleotide (NADH) and flavin adenine dinucleotide (FADH_2_) [73,74]. Electrons from reduced cofactors (NADH and FADH_2_) of tricarboxylic acid cycle and β-oxidation of fatty acids transfer electrons to complexes I and II before traversing complexes III and IV, result in active transport of H^+^ protons from the mitochondrial matrix across the inner mitochondrial membrane to create an electrochemical gradient [73,75,76], which is harnessed by ATP synthase to produce ATP [73,75,76]. Most of the electrons are completely consumed during this process, but some electrons leak from complexes I and III of the ETC and return to the mitochondrial matrix without ATP synthase. These electrons react with oxygen to produce superoxide and/or hydrogen peroxide [64,70,71]. Although the superoxide anion is not a particularly powerful oxidant, it serves as a potent precursor to the majority of other ROS and a proliferator of numerous oxidative chain reactions. Brown fat can counteract ROS formation through uncoupling mechanisms in the ETC of mitochondria, which is abundant in these cells [77] (Figure 7). Uncoupling protein facilitates the movement of protons from the inner membrane space to the mitochondrial matrix that does not couple during the ATP synthesis [78,79,80]. One of the main mechanisms by which brown fat neutralizes ROS and inflammation is through thermogenesis via uncoupling of mitochondrial respiration in the ETC [65]. Carbon monoxide has an uncoupling effect on mitochondrial respiration by activating mitochondrial BK_Ca_ channels (Ca^2+^-activated K^+^ channels), an uncoupling protein [81,82,83]. Other uncoupling proteins such as uncoupling proteins 1–3 (UCPs) and adenine nucleotide translocases (ANTs) are also activated by ROS to allow thermogenesis and gluthathione modulation, which lowers ROS formation and counteracts oxidative stress [81]. The UCP-1 causes a proton leak across the inner membrane of the mitochondria by converting the electrochemical energy into heat. Brown fat adipocytes degrade electrochemical chemical energy, and subsequently energy of the substrate is oxidized and converted into heat [78,79,84]. Uncoupling oxidative phosphorylation decreases ROS by lowering the inevitable oxidizing species formed by mitochondrial respiration in the ETC [81,83]. ROS are increased in obese and diabetic patients because this population has a higher white fat-to-brown fat ratio, which contains fewer mitochondria to perform thermogenesis [71,77] (Figure 6).

The combination of increased ACE-2R on adipocytes along with elevated levels of insulin, leptin, and acyl moieties allows COVID-19 to create the perfect “cytokine storm” leading to severe ROS formation which destroys mitochondria, especially in patients with abundant white fat cells (Figure 7). In severe cytokine storm you have inducible nitric oxide synthetase (iNOS), NO which will convert superoxides to peroxynitrite which is directly toxic to DNA and the vascular endothelium. The severity of the cytokine storm will inhibit HO-1. In addition, the uncoupling of mitochondrial enzymes will further increase ROS production. Increasing HO-1 levels decrease these inflammatory cytokines and iron toxicity in obesity. [68] This along with longer GT allele HO-1 polymorphisms lead to even more ROS formation as HO-1 levels in these individuals are lower at baseline with less inducibility, and therefore, lower CO levels are produced from the degradation of heme [52]. Moreover, adipocyte specific HO-1 gene expression is effective in restoring vascular function, insulin sensitivity, the conversion of white adipocytes to brown adipocytes [90], and improved mitochondrial function [91].

## 7. Clinical Presentation

Patients can be mildly symptomatic to severely ill with multi-organ damage/failure, multifocal pneumonia with acute respiratory distress syndrome (ARDS), rhabdomyolysis, anemia with increased oxygen requirements, and elevated liver enzymes. Most commonly, they will have leukopenia and lymphopenia, with a reversal of the CD4/CD8 ratio [92]. Elevated C-reactive protein (CRP) and ferritin levels suggest an impending cytokine storm [93]. Elevated D-dimers suggest the likelihood of deep vein thrombosis and venous thromboembolism (DVT/VTE) and anticoagulation must be considered [94,95]. COVID-19 patients can present with acute kidney injury complicated by acute renal failure, nephrotic range proteinuria, and/or electrolyte derangements [96]. Older patients have comorbid conditions like hypertension and diabetes [41]. Younger patients have more pronounced obesity and the chronic inflammatory state that it implies [60].

The organs mainly affected by COVID-19 infection includes the lungs (i.e., the endothelium of the pulmonary vasculature), kidneys, liver, and arterial endothelium (as evidenced by a prothrombotic state); all of which contain high concentrations of ACE-2Rs known to provide a binding site to COVID-19 to infect cells. There is concern that patients with hypertension on ACE inhibitors and angiotensin II receptor blockers (ARB’s) might have an increased risk of infection [97]. Since these drugs may upregulate ACE-2 expression, are they more susceptible to the SARS-CoV-2 virus that causes COVID-19? This continues to remain controversial, but current recommendations are to continue these drugs as indicated [97]. New articles published in the New England Journal of Medicine showed no evidence of harm. All were observational studies but all negate stopping these medications on patients who are currently taking them [98]. Evidence was presented in a recent JAMA article that ACEI/ARB therapy is not associated with increased risk of disease or severity of illness [99].

COVID-19 patients present with fever, headache, sore throat, cough, shortness of breath, and less commonly, myalgias, diarrhea, and nausea or vomiting [92]. Furthermore, the principal laboratory derangements seen in COVID-19 patients includes anemia, lymphopenia, elevated ferritin, elevated liver enzymes AST (aspartate transaminase) and ALT (alanine aminotransferase), elevated CRP (c-reactive protein), elevated D-dimer, and thrombocytopenia [100,101,102]. Initial imaging findings typically reveal bilateral infiltrates, unilateral infiltrates, or pleural effusion [100,101,102].

The overall clinical presentation of COVID-19 patients is consistent with the predilection of the virus to mainly affect cells with high ACE-2 receptors, likely as a result of the ability of the virus to decrease the amount of functional hemoprotein in these cells. These hemoproteins include hemoglobin, myoglobin, NO synthase, cytochromes within the electron transport chain (ETC), CYP enzymes, and catalase. Given that hemoprotein by definition contains heme, thus providing COVID-19 a substrate to bind to, COVID-19 has the potential to cause serious malfunction in all of the metabolic pathways in which these proteins are essential for normal function [7].

The basis for COVID-19 causing morbidity and mortality is severe systemic inflammation. This is the result of increased heme and free iron production, impairment of intracellular hemoprotein function, decreased levels of HO-1, and downregulation of HO-1 gene expression. The basis of current research to treat COVID-19 has focused on direct inhibition of viral replication and the inflammatory response.

## 8. Cytochrome 2D6 Genetic Polymorphisms COVID-19

Chloroquine was first introduced in 1934. Hydroxychloroquine/chloroquine are both 4-aminoquinolones approved in the treatment of Malaria, Systemic Lupus Erythematosus, and Rheumatoid Arthritis [103]. The FDA approved these drugs for “emergency use” in hospitalized patients with COVID-19 in March of 2020. Many proposals to use these drugs for coronavirus are based on in vitro and animal studies where they proved beneficial, the use of chloroquine was based on evidence of it being a potent inhibitor of SARS coronavirus infection and spread [104]. The data for the prevention and treatment of severely ill patients have so far been disappointing. During the MERS epidemic, 4-aminoquinolones were shown to affect glycosylation of ACE-2 receptors, which is used by COVID-19 to enter cells [104].

There is comprehensive literature on the genetic polymorphisms of cytochrome P450 (CYP) 2D6 that affects metabolism of drugs that use this pathway, which includes almost 50% of the drugs currently in use [105]. These polymorphisms can increase the metabolism (ultrarapid metabolizers) that might decrease their effectiveness or markedly decrease their metabolism (slow metabolizers) resulting in toxicity [106]. Slow metabolizers are more common. Slow metabolizers allow toxic levels of drugs like hydroxychloroquine to accumulate resulting in increased cardiac complications like prolonged QTc syndrome, with increased risk of cardiac death, particularly in obesity and diabetes, where ACE-2 is upregulated (Figure 7) due to an increase of white adipocytes and a reduction in brown adipocytes.

Polymorphism of CYP2D6 is higher in African Americans and Asians [107,108,109], disproportionately affected by this disease. One study in Lupus patients in Korea showed significant variability in hydroxychloroquine levels due to CYP2D6 polymorphisms [110]. Another study in discoid lupus found that 39% had either poor results from fast metabolism or poor outcomes related to toxicity from slow metabolism [111]. This accounts for the differences in clinical outcomes with this medication.

There are multiple accounts of these genetic polymorphisms resulting in resistant strains of malaria because of abnormalities in metabolism [112,113,114]. These same gene polymorphisms affect heart failure patients since the CYP 2D6 gene is responsible for the metabolism of metoprolol [115,116]. This polymorphism affect many other drugs such as Isoniazid (INH), barbiturates, omeprazole, selective serotonin reuptake inhibitors (SSRI’s), sulfasalazine, hydralazine, etc. [75].

Patients with genetic polymorphisms of the CYP2D6 and the HO-1 GT allele make treatment and prognosis challenging. Carriers of these different polymorphisms will respond well to medications and have less risk of developing complications from COVID-19 such as ARDS and multiorgan failure, while others will develop toxic drug levels and multiorgan failure [106] (Figure 4). This may explain the unpredictability clinicians are observing with COVID-19 patients and variable results regarding 4-aminoquinolones. More recent data suggests that 4-aminoquinolones showed no benefit over the standard of care and patients were at higher risk of developing cardiac arrest in the treatment group, especially when combined with Azithromycin [117,118].

## 9. Therapeutic Strategies-COVID-19

### 9.1. Antivirals

Remdesivir, a CYP3A4 substrate, is an anti-viral agent developed to treat Ebola but proved ineffective [119]. It also showed promise in SARS and MERS [120,121]. The drug is currently being used in clinical trials to treat COVID-19. It is an adenosine analogue that inserts itself into the RNA virus, terminating its replication. It has been the most promising antiviral agent currently tested (in two phase III randomized clinical trials) against COVID-19. CYP3A4 transcription is affected by cytokines and glucocorticoids, downregulated by the acute phase reactant IL-6 and upregulated by dexamethasone [122]. As a result, concomitant use of steroids along with Remdesivir may lead to lower drug levels of Remdesivir in COVID-19 patients with severe inflammation, and therefore higher IL-6. This may be the reason behind reports of adverse events in Remdesivir recipients versus placebo [123]. The anti-influenza RdRp inhibitor favipiravir is also being clinically evaluated for its efficacy in COVID-19 patients [124]. Favipiravir is undergoing phase II clinical trials in China and Thailand with reports of efficacy when combined with interferon [125]. Favilavir, another antiviral, was the first approved COVID medication in China. In a clinical trial of 70 patients, the drug reportedly showed efficacy in treating the disease with minimal adverse effects [126]. Another antiviral agent that was tested against COVID-19 was the protease inhibitors Lopinavir/ritonavir (LPV/RTV). This drug combination was found to reduce mortality rate and lead to a milder disease course during the open clinical trial in the 2003 SARS outbreak [127,128]. However, LPV/RTV alone has not proved to be superior to the standard of care in COVID-19 patients [129]. Interestingly, LPV is a substrate of CYP3A4, while RTV is a potent inhibitor of CYP3A4 [130]. Is it possible that RTV potentiates the effects of LPV by increasing drug levels of LPV? Further research needs to be done to evaluate this. Current in vitro studies are examining LPV/RTV and interferon beta against COVID-19 [131].

### 9.2. Cytokine Inhibitors

IL-6 inhibitors, such as Tocilizumab, are currently being tried as a potential treatment for COVID-19 patients to reduce inflammation in the lungs and prevent progressive damage [132]. Results thus far have shown benefit in the treatment group versus the standard of care and are currently in phase III clinical trials as of April 15th. There are reports of Gram-negative sepsis with the use of this drug [133]. Tocilizumab has the potential to affect the expression of several CYP enzyme polymorphisms including CYP3A4 and CYP2D6 [134].

### 9.3. Antiretrovirals, Monoclonal Antibodies, Convalescent Plasma, and Corticosteroids

Leronlimab, the CCR5 (chemokine receptor 5) inhibitor, is currently entering Phase II clinical trials. This is an antiretroviral agent used to prevent the virus from entering the CD4 cell by blocking the CCR5 receptor [135]. CCR5 inhibitors are also being studied and have been found to significantly reduce IL-6 and TNF-a with a lower toxicity profile than Tocilizumab [136]. It has an excellent safety profile, used extensively in triple negative breast cancer and in human immunodeficiency virus (HIV) treatment [137,138]. There is a granulocyte-macrophage colony-stimulating factor (GM-CSF) monoclonal antibody, Lenzilumab, which is being tested (Phase III clinical trial) for prevention of respiratory failure and/or death in COVID-19 patients [139]. Convalescent plasma has been administered to patients who are seropositive for acute COVID-19 and hypoxic, which lead to undetectable viral load and improved oxygenation in 3 days after one dose in non-intubated patients [140]. Convalescent plasma has been shown to reduce hypoxia in intubated patients [140]. The adverse effects associated with plasma transfusions include the risk of TRALI, TACO, and anaphylactic reactions [141].

Immunosuppression therapy is being examined to counteract the severe systemic inflammatory reaction caused by SARS-COV-2 infection. One of the modalities being considered is corticosteroids. Corticosteroids should only be used in COVID-19 induced lung injury in the setting of a clinical trial [142]. There is data that concludes harm in using corticosteroids in COVID-19 patients, while other studies have been inconclusive or biased with confounding factors [142]. Some studies were noted to indicate benefit in COVID-19 patients with a low-to-moderate dose [142]. Nonetheless, the use of corticosteroids remains controversial given the methodological limitations in the available evidence [142]. A more recent randomized clinical trial (RCT) published in the UK involving 2100 patients in the treatment arm has shown promising results with the use of dexamethasone [143]. In the patients who were critically ill on ventilators secondary to COVID-19, the treatment group showed a decrease in mortality by 1/3 vs. the standard of care [143]. Furthermore, those who required supplemental oxygen but were not intubated showed a 20% decrease in mortality rates [143]. Given the feasibility and cost effectiveness of dexamethasone, it has been approved by the UK government for use in COVID-19 patients. These results emphasize that the inflammatory storm caused by COVID-19 could present a critical therapeutic target to mitigate the deleterious effect of the virus. In summary, there has been no magical therapy to treat COVID. The search remains for an approach that combines antiviral therapy with an approach to reducing the inflammatory cytokines.

## 10. Heme Arginate-HO-1 Levels—Inflammation

Heme arginate increases HO-1 levels by inhibiting delta-aminolevulinate synthase (ALAS-1/2), the rate limiting enzyme in the eight steps of the heme synthetic pathway [55]. Heme arginate also promotes glucagon release, which results in an increase of 2.3-fold in HO activity [144]. Previous studies have shown that increasing levels of HO-1 with heme arginate can improve inflammation and viral infections [145,146,147,148]. Sickle cell patients, for example, are in a pro-inflammatory state [149]. HO-1 upregulation reduced oxidative stress in the kidney in sickle cell disease [150]. Heme arginate, which is an upregulator of HO-1, was studied in this population and found to prevent dysfunctional hemoprotein formation, while increasing the ability of ferritin to chelate iron [150]. Increased levels of HO-1 play an essential role in the induction of ferritin synthesis as a result of increased levels of free iron from degradation of heme, which acts as antioxidant protection [151,152,153]. Increasing levels of HO-1 decreases lipid peroxidation, red blood cell (RBC) sickling, and heme content in the kidney [150]. Heme arginate also increases Nitric Oxide (NO) while decreasing adhesion molecules in HIV infected stem cells leading to a decrease in the number of HIV copies in cells [145,146,147]. This inhibition of HIV replication requires decreased adhesion, increased levels of antioxidants, and inhibition of viral transcriptase enzymes, all of which was accomplished by administration of azidothymidine (AZT)-Heme arginate [145,146,147]. This combination was successful even in an AZT resistant HIV strain [147]. HO-1 upregulation by heme arginate decreased dysfunctional hemoprotein, which resulted in HO-1 upregulation attenuating the EBOLA virus (EBOV) transcription/replication leading to a decrease in viral particles [148]. Heme arginate is well known to decrease heme production and has been used extensively to treat porphyria patients. The resultant increase in HO-1 reduces vasoconstriction [154,155], hepcidin [156], and inflammation with the potential ability to prevent the cytokine storm [55]. This is especially important in patients who are predisposed to “the perfect cytokine storm” from COVID-19, including those with the longer GT allele HO-1 polymorphism, abundant white adipose cells (in obesity and diabetes), and those with CYP2D6 polymorphisms that lead to supra- or sub-therapeutic drug levels. There are a number of naturally occurring substances that are capable of upregulating HO-1. These include Thymoquine (black seed oil) in combination with Omega-3 that was shown to protect against obesity induced oxidative stress, improve insulin sensitivity and convert white fat to beige fat in a murine model of obesity [157]. In addition, pomegranate seed oil upregulated HO-1, improved mitochondrial function, and attenuated hepatic steatosis/fibrosis in a murine model of obesity [158]. It has previously been reported that HO-1 was upregulated in response to other naturally occurring substances such as resveratrol, curcumin, statins, and aspirin [19].

## 11. ACE Genetic Polymorphisms

**Figure 8:**
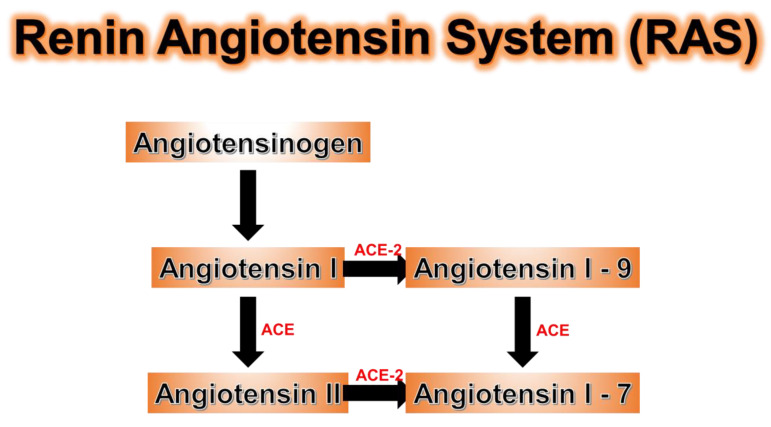
ACE-2 is needed to convert pro-inflammatory angiotensin II (ANG-II) to anti-inflammatory ANG (1-7) in the renin-angiotensin system (RAS). As COVID-19 competes for ACE-2, less ANG-II is converted to ANG (1-7) which leads to more inflammation. Furthermore, individuals with ACE genetic polymorphism DD have higher levels of ACE activity which promotes more formation of ANG-II making individuals with this polymorphism susceptible to even greater inflammation. Individuals with the ACE polymorphism have the lowest ACE activity, which leads to lower levels of ANG-II and less inflammation.

ACE levels are under genetic control and much research had been dedicated to understanding polymorphisms of the ACE genes [159]. Individuals with ACE gene polymorphisms are susceptible to the severe inflammation induced by COVID-19. Angiotensinogen is produced in the liver and is upregulated in adipocytes in obesity, while adipocyte angiotensin deficiency can prevent hypertension in the obese populations [160,161,162]. The major role of ACE-2 in the renin-angiotensin system (RAS) is in the conversion of angiotensin II (ANG-II) to angiotensin 1-7 (AT 1-7) (Figure 8). ANG-II promotes obesity associated metabolic diseases, oxidative stress, and inflammation in the lungs, vascular endothelial cells, and kidney epithelial cells [159,163]. In contrast, AT 1-7 promoted cardiovascular (CVS) protection along with anti-inflammation, anti-proliferation, and anti-fibrosis in organs with high ACE-2 concentrations [163]. ACE-2 is present in the heart, lungs, kidney, testes, and adipose tissue with the highest levels in adipocytes [164,165]. ACE-2 is upregulated in adipose tissue in obesity and diabetes, with an increase in the ACE-2 receptors in white adipose cells, but more importantly, a major increase in the number of white adipocytes in obesity accounts for the increase in total ACE-2 receptors [163]. Since the spike protein of the COVID-19 virus enters the cell via the ACE-2 receptor, it remains unclear if this is the reason why obesity, with a significant upregulation of ACE-2 receptors, increases the risk of significant morbidity and mortality. Genetic polymorphisms exist for ACE-2 just as in ACE, and ACE activity has been well studied in individuals with different genetic polymorphisms [166]. Those with the DD genotype had higher ACE activity levels than those with the ID genotype (intermediate ACE activity), which was associated with higher ACE activity than those with the II genotype (least ACE activity) [166]. Since ACE is responsible for converting inactive ANG-1 to proinflammatory ANG-II, those with higher ACE levels are at higher risk of developing severe inflammation secondary to COVID-19 infection [166] (Figure 8).

## 12. Conclusions

The main drugs currently being used to treat COVID-19 patients such as the 4-aminoquinolones, azithromycin, and remdesivir all decrease heme production, in part, by inhibiting CYP enzyme induction, thus decreasing the demand for heme. These drugs also display interactions with the CYP system, which makes maintaining safe effective therapeutic drug levels a challenge. Heme arginate is a more powerful upregulator of HO-1 and inhibitor of ALAS, lowering heme levels substantially. Furthermore, Heme arginate is able to accelerate reactions mediated by CYP2D6 and CYP3A4 [108,110]. Future combination of Heme arginate and LPV/RTV or drugs that are not metabolized by CYP2D6/3A4 might have a synergy as an efficacious dual drug-therapy to counteract the severe inflammation caused by COVID-19.

Obesity is a chronic inflammatory disease manifested by an increase in cytokine levels at baseline, increasing the risk of a “cytokine storm” in obese patients. Obese individuals have many more ACE-2 receptors on the surface of white necrotic adipocytes and have many more white adipocytes in total than the number of brown adipocytes of their lean counterparts. COVID-19 causes severe systemic inflammation and downregulation of HO-1, already downregulated in the chronic inflammatory state of obesity. The clinical course of the disease has been challenged by the interaction of three genetic polymorphisms, all affected by COVID-19: first is the CYP2D6 enzyme system, second is the HO-1 anti-inflammatory gene, and third the ACE and ACE-2 enzyme systems. Effective therapy must include anti-viral drugs to halt viral replication. The second must involve the upregulation of the heme oxygenase system to reduce ROS and the severe inflammation caused by the cytokine excess. The anti-inflammatory and antioxidant approach should be essential to our armamentarium until we have an effective vaccine.

## Figures and Tables

**Table 1:** This represents a list of functional hemoproteins and the clinical scenarios caused by dysfunction of these hemoproteins. Key: AKI = acute kidney injury, ARF = acute renal failure, CHF = congestive heart failure, AOCI = anemia of chronic inflammation.

Consequences of Hemoprotein Malfunction/Deficiency			
Hemoprotein	Deficiency Consequence	Clinical Manifestation	Main Organs Affected
Catalase	↑ ROS formation	Tissue damage	High [ACE-2R] organs
Cytochrome of the ETC	↓ Aerobic respiration ↑ Apoptosis	Tissue damage	High [ACE-2R] organs
NO synthetase	↓ vessel hemostasis ↓ smooth M. relaxation ↑ cardiac contractility ↓ regulation of renal hemodynamics, sodium regulation, and tubuloglomerular feedback	Prothrombotic state Vasoconstriction CHF/tachycardia AKI/ARF	High [ACE-2R] organs
Myoglobin	↑ Rhabdomyolysis	Rhabdomyolysis Acute kidney injury Electrolyte derangement	Skeletal muscle
Hemoglobin	↓ O_2_ and CO_2_ affinity ↓ Production	Prolonged and increasing O_2_ requirements Anemia (AOCI)	RBCs
All cells with porphyrin in membrane	No deficiency. Allows COVID-19 to bind with high affinity leading to infection	Progressive tissue damage, especially as virus continues to replicate within the cell	Low [ACE-2R] organs

## Abbreviations

ACE	angiotensin converting enzyme
HO	heme oxygenase
HIV	human immunodeficiency virus
SARS	acute respiratory distress syndrome
MERS	middle East respiratory syndrome
ROS	reactive oxygen species
NO	nitric oxide
cAMP	cyclic adenosine monophosphate
TMPRSS-2	transmembrane serine protease 2
ETC	electron transport chain
OH^−^	hydroxide
p38 MAPK	p38 mitogen-activated protein kinases
PBGD	porphobilinogen deaminase
ALA	delta-aminolevulinic acid
HMB	hydroxymethylbilane
ETC	electron transport chain
AKI	acute kidney injury
ARF	acute renal failure
CHF	congestive heart failure
AOCI	anemia of chronic inflammation
Ox-HDL	oxidized high-density lipoprotein
VLDL	very low-density lipoprotein
IL	interleukins
TNF	tumor necrosis factor
NADH	nicotinamide adenine dinucleotide
FADH_2_	flavin adenine dinucleotide
ANTs	adenine nucleotide translocases
UCP	uncoupling proteins
INOS	inducible nitric oxide synthetase
ALT	aspartate transaminase
AST	alanine aminotransferase
CRP	c-reactive protein
LPV/RTV	Lopinavir/ritonavir
RTC	randomized clinical trial
AZT	azidothymidine
